# Post-burn Inguinoperineal Contracture With Intercrural Adhesion: A Case Report

**DOI:** 10.7759/cureus.107589

**Published:** 2026-04-23

**Authors:** Actheinay Cruz Cobo, Yelka Matos Furones, Luis F Gonzalez Vazquez, Ruben E Diaz Samada, Mara I Romero, Chloe M Asante

**Affiliations:** 1 Plastic and Reconstructive Surgery, Medicina Integra Specialty Clinic, Cancún, MEX; 2 North Georgia Clinical Research/Neurology, Alcanza Clinical Research, Woodstock, USA; 3 Anesthesiology, Geisinger Medical Center, Danville, USA; 4 Internal Medicine, Saturnino Lora General Hospital, Santiago de Cuba, CUB; 5 Surgery, HCA Florida Memorial Hospital, Jacksonville, USA

**Keywords:** direct closure, inguinoperineal burn, intercrural adhesion, post‑burn contracture, reconstructive surgery, scar release

## Abstract

Post‑burn inguinoperineal contractures are rare but can lead to severe functional impairment, anatomical distortion, and significant psychosocial burden when not treated promptly. We report the case of a 25‑year‑old woman with a nine‑year‑old inguinoperineal burn initially managed with traditional methods and without specialized medical care. She presented with a severe contracture and extensive intercrural adhesion that markedly limited thigh abduction and distorted the external genital anatomy, while the remainder of her physical examination was unremarkable. Surgical management consisted of complete excision of the fibrotic tissue, full release of the contracture, sharp division of the intercrural adhesion, and anatomical reconstruction of the inguinal folds, followed by tension‑free direct closure. Standard perioperative antibiotic prophylaxis was administered due to the inguinoperineal location and extent of the procedure, with no infectious complications observed. The postoperative course was uneventful, with appropriate healing at the seven‑day evaluation and progressive suture removal on postoperative days 12 and 14. Subsequent follow‑up at six weeks and three months demonstrated normal scar evolution, absence of dehiscence, retraction, or recurrent contracture, and stable preservation of the functional range of motion achieved after surgery. This case highlights that even long‑standing post‑burn contractures can be effectively corrected through complete surgical release and tailored anatomical reconstruction, and that direct closure may represent a safe and feasible option in settings where advanced reconstructive techniques are not available.

## Introduction

Post‑burn scarring arises from the normal wound‑healing response following thermal injury; however, in many cases, it may evolve pathologically, leading to excessive collagen deposition, tissue retraction, and progressive loss of elasticity. These pathological scars can progress to contractures, particularly in flexural regions where constant motion, moisture, and mechanical tension predispose to severe functional impairment. Globally, burn injuries account for an estimated 11 million cases annually, with the highest burden occurring in low‑ and middle‑income countries [1,2]. Inguinoperineal post‑burn contractures are rare but reported more frequently in regions with limited access to early burn care, including parts of sub‑Saharan Africa [3]. In Djibouti, epidemiological data are lacking, and no studies specifically describe the incidence of inguinoperineal contractures or intercrural adhesions; therefore, the local burden of these conditions remains unknown [4].

Several reconstructive approaches have been described for the management of post‑burn contractures in the inguinoperineal region, including complete scar excision followed by split‑thickness or full‑thickness skin grafting, Z‑plasties, local advancement flaps, regional fasciocutaneous flaps, and, in complex cases, myocutaneous flaps [5-7]. Regardless of the method, the cornerstone of successful reconstruction is complete release of the retractile scar tissue to restore anatomical relationships and functional mobility [6,7]. In the present case, the patient was treated in a hospital with limited reconstructive resources, where advanced flap options were not available. Under these constraints, complete surgical release followed by direct closure was selected as a pragmatic approach based on the resources available.

## Case presentation

A 25‑year‑old African woman from Djibouti presented with a long‑standing post‑burn inguinoperineal contracture resulting from a scald injury caused by boiling oil. She had no history of prior surgical attempts to correct the deformity. Baseline assessment showed preserved urinary function and a normal menstrual pattern; however, she reported recurrent vaginal infections due to difficulty maintaining adequate perineal hygiene secondary to the severe anatomical distortion and partial occlusion caused by the intercrural adhesion. She also described complete inability to engage in sexual intercourse, substantial limitations in daily activities requiring lower‑limb separation, and progressive discomfort during ambulation. These functional impairments, together with the chronic deformity, contributed to significant psychological distress and altered body image.

**Figure 1:**
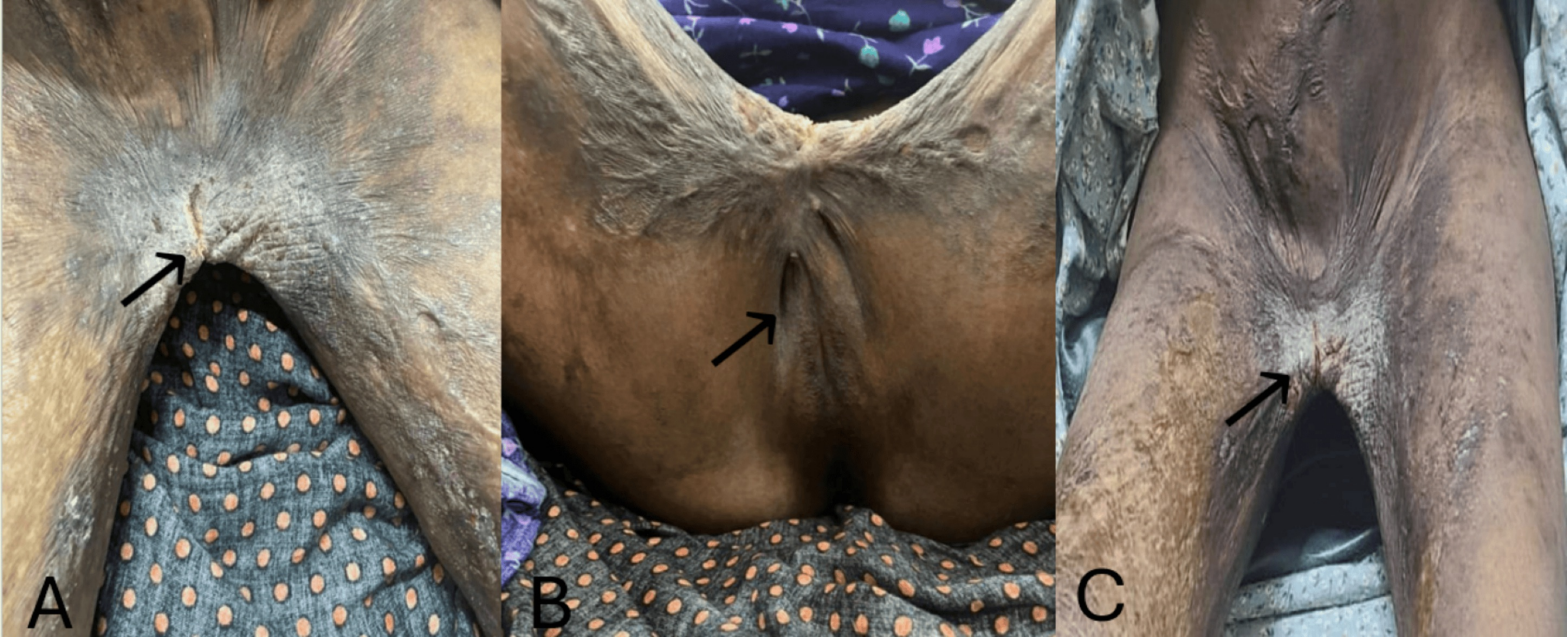
Preoperative findings. (A) Severe distortion of the external genital anatomy secondary to an inguinoperineal post‑burn contracture (black arrow). (B) Intercrural adhesion with obliteration of the inguinal folds and loss of normal anatomical contours (black arrow). (C) Dense retractile scar bands causing limitation of thigh abduction and narrowing of the perineal space (black arrow).

Focused examination revealed a severe bilateral inguinoperineal contracture with extensive intercrural adhesion fusing the medial thighs. The external genital anatomy was markedly distorted, with partial obscuration of the labia majora and limited visualization of perineal landmarks. Thigh abduction was restricted to an estimated total range of approximately 15° (inter‑knee distance of 18 cm), preventing adequate exposure of the vulvar structures and compromising normal functional positioning (Figure 1).

Surgical technique

The patient underwent a comprehensive preoperative evaluation and reconstructive planning before being transferred to the operating room, where she was positioned in the supine position with moderate abduction of both lower limbs to allow adequate exposure of the inguinoperineal region and the medial aspects of the thighs. After administration of regional anesthesia, perioperative antibiotic prophylaxis was initiated following the institutional protocol for clean‑contaminated procedures in the perineal region, using a standard regimen of intravenous ceftriaxone combined with metronidazole to mitigate the risk of surgical site infection. This protocol was maintained for 48 hours postoperatively due to the inguinoperineal location and the extent of the surgical procedure, with no infectious complications observed.

Preoperative markings were then performed over the retracted scar bands and planned resection areas, carefully delineating the segments to be released while preserving viable pubic tissue to facilitate subsequent anatomical reconstruction of the inguinal folds. Wide asepsis and antisepsis were performed, encompassing the lower abdomen, pubic region, bilateral groins, anterior perineum, and medial thighs, followed by placement of sterile drapes to ensure optimal exposure and intraoperative maneuverability.

The procedure began with incisions along the previously marked areas, followed by meticulous layer‑by‑layer dissection and complete excision of the fibrotic bands responsible for the contracture. Release was performed progressively, separating the medial thighs and restoring the intercrural space, with rigorous hemostatic control throughout. During dissection, viable pubic tissue was identified and preserved to avoid unnecessary resection and ensure adequate coverage for reconstruction.

**Figure 2:**
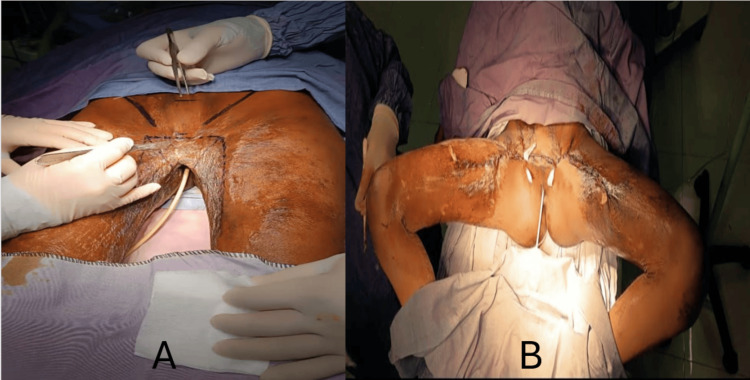
Intraoperative reconstruction and closure. (A) Preoperative surgical markings delineating the retractile scar bands and planned release lines, aimed at achieving complete contracture correction and facilitating anatomical reconstruction of the inguinal folds. (B) Immediate postoperative result following direct layered closure, demonstrating restoration of the inguinoperineal anatomy, redefinition of the inguinal folds, and re‑establishment of intercrural separation with adequate thigh abduction.

Once full release was achieved, intraoperative functional assessment was performed by controlled abduction and flexion of both thighs to confirm restoration of the range of motion and absence of residual tension or restrictive bands. Reconstruction of the inguinal folds was then performed by repositioning and anchoring the preserved pubic tissue to the abdominal wall, redefining the natural creases and reducing the risk of secondary retraction. After confirming adequate mobility and absence of excessive tension, direct layered closure of the raw areas was performed with careful tissue approximation. Final hemostasis was verified, the surgical field was irrigated, and an appropriate dressing was applied, completing the procedure with anatomical restoration of the inguinoperineal region, re‑establishment of intercrural separation, and immediate recovery of lower‑limb mobility (Figure 2).

Postoperative course

**Figure 3:**
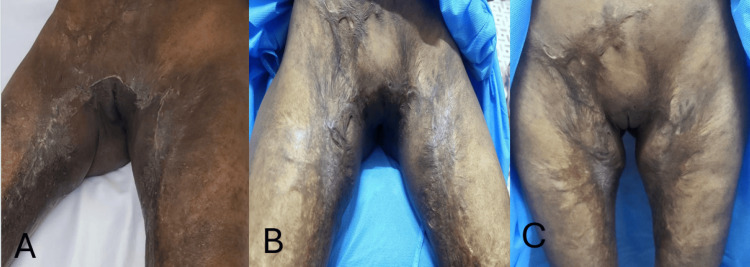
Postoperative evolution. (A) Fourteen‑day postoperative view showing appropriate wound healing, a stable inguinal fold contour, and preserved intercrural separation. (B) Six‑week follow‑up demonstrating favorable scar maturation without evidence of dehiscence or secondary contracture. (C) Late functional and anatomical outcome with preserved range of motion, adequate thigh abduction, and a stable inguinoperineal reconstruction.

The patient experienced an uneventful postoperative recovery. At the seven‑day follow‑up visit, the surgical site demonstrated appropriate healing with no signs of infection, dehiscence, or local complications. The reconstructed inguinal folds maintained their contour, and the separation between the medial thighs remained stable. Sutures were removed progressively on postoperative days 12 and 14 in an alternating pattern, with no wound‑related complications. After suture removal, outpatient evaluations at six weeks and three months showed normal scar maturation without dehiscence or retraction, and a postoperative range of motion consistent with the early functional gains achieved after surgery (Figure 3).

**Figure 4:**
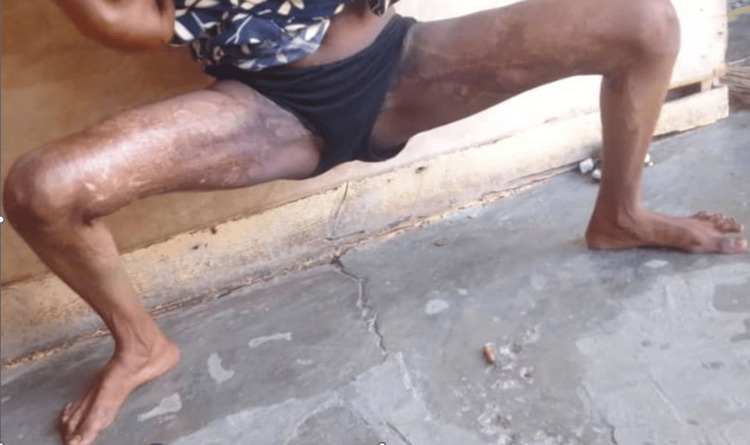
Long‑term postoperative outcome at 36 months, demonstrating stable anatomical restoration with thigh abduction reaching 85° (inter‑knee distance of 55 cm).

At the 36‑month follow‑up, the patient demonstrated stable and sustained anatomical restoration. Thigh abduction reached a total of 85° (inter‑knee distance of 55 cm), representing a 70° improvement from the preoperative baseline. Functionally, she exhibited a normal gait pattern and complete resolution of her previously recurrent vaginal infections following the restoration of adequate perineal hygiene conditions. These long‑term outcomes are illustrated in Figure 4.

Ethical approval

Written informed consent was obtained from the patient for publication of this case report and accompanying images.

## Discussion

Pathological scarring encompasses a spectrum of aberrant wound‑healing responses that may result in functional, aesthetic, and anatomical impairment. Among these, hypertrophic scars are characterized by raised, erythematous tissue confined to the boundaries of the original wound and typically arise in areas subjected to high tension or delayed healing [8]. Although common in burn survivors, our patient did not exhibit hypertrophic scarring; instead, the deformity was dominated by dense retractile fibrosis rather than elevated scar proliferation.

Keloids, in contrast, extend beyond the original wound margins and demonstrate uncontrolled collagen deposition with a high recurrence rate [9]. These lesions are more prevalent in individuals with higher‑risk skin phototypes and in anatomical regions such as the chest and shoulders. Despite the patient’s higher‑risk phototype, no keloidal characteristics were identified.

Atrophic scars, defined by dermal thinning and loss of subcutaneous tissue, are typically associated with acne or varicella rather than burns [10]. They present as cutaneous depressions or irregularities but do not produce the mechanical restriction observed in this case. Pigmentary scars, whether hyperpigmented or hypopigmented, result from melanocyte dysfunction following inflammation or dermal injury [11]. Although pigmentary alterations are common in burn survivors, they do not cause anatomical distortion or functional limitation and were not a predominant feature in this patient. Mixed scars, which combine two or more pathological patterns, may occur in extensive burns [12], but the clinical presentation here was clearly dominated by a single pathological entity: a severe contracture.

Contracture scarring represents the most functionally disabling form of pathological scarring. It results from progressive tightening and alignment of collagen along lines of tension, particularly in flexural regions [3,5,7]. These lesions restrict mobility, distort anatomical landmarks, and may compromise essential functions such as ambulation, hygiene, and sexual activity. In the inguinoperineal region, contractures are especially debilitating due to constant movement and inherent moisture [3,6]. Our patient exhibited the classic features of a long‑standing post‑burn contracture, including extensive intercrural adhesion, near‑complete loss of thigh abduction, and distortion of the external genitalia. No additional pathological scar types were present, confirming that the deformity was attributable exclusively to a severe contracture.

Multiple surgical strategies have been described for the management of post‑burn contractures, including Z‑plasty, local tissue rearrangement, split‑ or full‑thickness skin grafting, regional fasciocutaneous flaps, and myocutaneous flaps for extensive defects [5,7,13,14]. The choice of technique depends on the severity of the contracture, tissue availability, and the reconstructive resources of the treating institution. In this case, complete release of the retractile scar bands provided sufficient tissue laxity to allow direct layered closure without tension. This approach offered a safe and effective solution within the constraints of the local healthcare setting and resulted in restoration of anatomical contours, improved functional mobility, and a favorable postoperative course.

Although contractures of this severity often require the introduction of new tissue, several context‑specific factors influenced the reconstructive strategy in this case. Local humidity and friction increased the risk of graft loss, making direct closure a safer option within our setting. Creating a rhomboid‑shaped defect allowed redistribution of tension toward areas with greater tissue laxity, while suprapannicular undermining facilitated the use of the region’s residual elastic reserve. Notably, the combination of healthy tissue with stable scar tissue provided durable long‑term stability. We acknowledge that inter‑knee distance and angular estimation are less precise than digital goniometry; however, they offer practical and objective metrics for documenting functional progression in resource‑limited environments.

Limitations

This report describes a single‑patient experience, which inherently limits the generalizability of the findings. Although a 36‑month postoperative photograph provides valuable long‑term confirmation of stable anatomical reconstruction and sustained functional recovery, the absence of serial clinical evaluations over that period limits the ability to assess subtle changes in scar maturation or late functional fluctuations. Additionally, the surgical strategy was shaped by the constraints of a resource‑limited setting, which may differ from the reconstructive options available in higher‑complexity centers. Despite these limitations, this case offers meaningful insight into the management of rare inguinoperineal post‑burn contractures and supports the effectiveness of complete release and direct closure when advanced techniques are not feasible.

## Conclusions

Post‑burn inguinoperineal contractures are uncommon but can lead to significant functional impairment, anatomical distortion, and psychosocial burden when not treated promptly. Early access to specialized burn care is essential to prevent the progression of deep scarring into severe deformities such as intercrural adhesion. In this patient, complete surgical release followed by tension‑free direct closure resulted in favorable anatomical and functional recovery. While this approach is not broadly generalizable, it may represent a feasible option in carefully chosen cases, particularly in resource‑limited settings where advanced reconstructive techniques are not available. Longer follow‑up and additional cases are needed to better define the reproducibility and long‑term safety of this strategy.
